# "Restoring Function": A Case Report on Using the Tongue Flap for Anterior Palatal Fistula Repair

**DOI:** 10.7759/cureus.90464

**Published:** 2025-08-19

**Authors:** A. Vijay Kumar, Ravali Sriram, G. Siva Prasada Reddy, Mohammed Darain Shahid, Kavya Rachana Chatharasi

**Affiliations:** 1 Oral and Maxillofacial Surgery, Annamaneni Vikas Rishith (AVR) Hospital, Hyderabad, IND; 2 Oral and Maxillofacial Surgery, Panineeya Mahavidyalaya Institute of Dental Sciences and Research Centre, Hyderabad, IND; 3 Oral and Maxillofacial Surgery, Panineeya Institute of Dental Sciences and Research Centre, Hyderabad, IND

**Keywords:** anterior palatal fistula, cleft palate repair, oro-nasal fistula, pedicled tongue flap, two-stage procedure

## Abstract

Orofacial clefts are some of the most frequent birth defects worldwide, posing a significant health challenge. Palatal clefts are particularly prevalent and can significantly affect a child's health and overall development. They result in communication difficulties and social withdrawal, feeding difficulties, and psychological issues. In this case report, we used anterior-based pedicled tongue flap for closure of palatal fistula as this method is favored due to the tongue's rich blood supply and potential for successful closure, even in complex cases.

## Introduction

"Palatal defects can significantly impair speech clarity, swallowing efficiency, and overall social interaction, leading to reduced quality of life." Tongue flaps are a reconstructive surgical technique that uses tongue tissue to fix deformities in the oral cavity, lips, and surrounding tissues. This treatment includes harvesting a part of the tongue, usually from the dorsal or lateral side, to form a vascularized tissue flap that may be transplanted to the defect site. Tongue flaps are categorized into pedicled and free flaps based on their function and usage. The tongue flap's strong vascular supply, ease of movement, and great healing potential make it a popular choice for treating surgical difficulties, especially when local tissues are limited or have previously been injured [1].

In the late 1800s, surgeons started exploring the usage of the tongue as a donor site to heal oral cavity defects. Harold Delf Gillies, a revolutionary plastic surgeon, coined the term "tongue flap" in the early twentieth century. During the 1950s and 1960s, plastic and reconstructive surgeons improved the procedure for tongue flaps. Conley [2] and Bakamjian [3] were among the first to demonstrate the effectiveness of tongue flaps in head and neck surgery. In 1963, the Surgical Department of the Dermatological Institute at the University of Guadalajara in Mexico began using tongue flaps to heal medium-sized palatal fistulas. Typically, the flap thickness is at least 3 mm, with a maximum of 5 mm at the base.

This treatment is commonly utilized in plastic surgery, oral and maxillofacial surgery, and otolaryngology to effectively restore abnormalities caused by cleft palate operations, oral cancer resections, and traumatic defect repairs, and is now being enhanced through improved vascular investigations and surgical procedures.

## Case presentation

**Figure 1:**
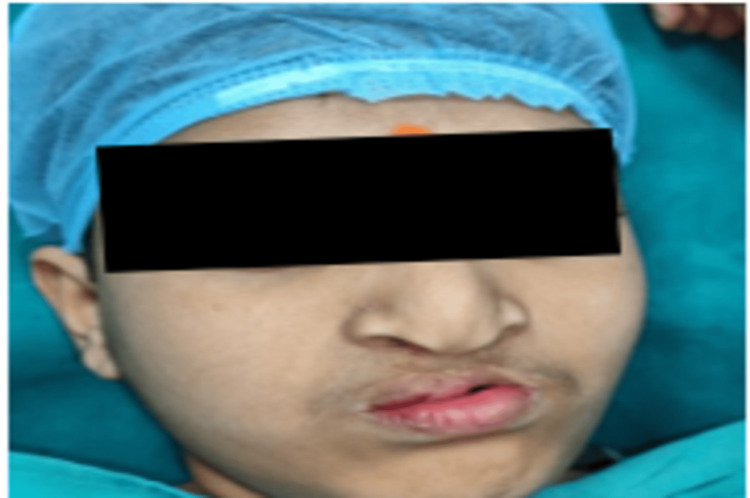
Front profile of the patient showing scarring of the upper lip, whistle deformity, inadequate bulk in the center of upper lip, and deficient lip height

A 16-year-old male child presented to the hospital with the primary complaint of a palatal defect and leakage of liquids through the nose. The patient's father gives a history of cleft lip and palate at birth (Veau IV-bilateral palate and alveolus). He underwent primary cleft lip and cleft palate repair. At the age of 13 years, the patient underwent treatment for a palatal fistula, which occurred as a complication of primary cleft palate closure. In spite of repair, the patient still had an anterior palatal fistula, for which he visited our hospital for further treatment. There was nasal regurgitation on taking fluids, and he also complained of speech difficulty. Extraoral examination showed scarring of the upper lip, whistle deformity, inadequate bulk in the center of the upper lip, and deficient lip height (Figure 1).

On clinical examination, two wedge-shaped oronasal defects were noted on either side of the midpalatal raphe, consistent with the patient’s history of bilateral cleft palate.

On the left side, a defect measuring approximately 3 × 1.8 cm was present in the anterior hard palate. It extended anteroposteriorly from the mesial aspect of tooth 11 onto the palate up to the distal aspect of tooth 25, and mediolaterally from the midpalatal region to about 1 cm short of the free gingival margin.

**Figure 2:**
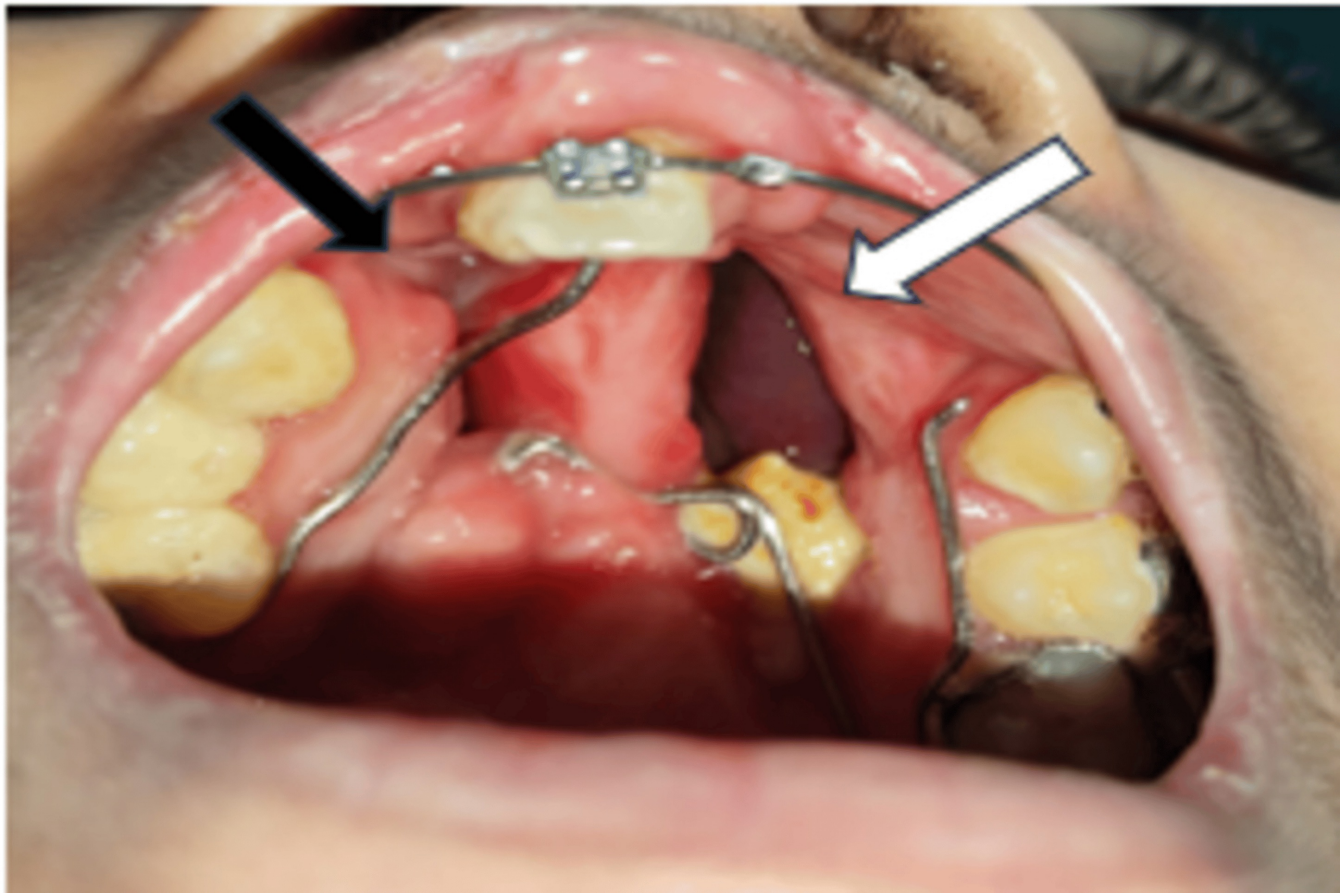
1- Fistula measuring about 3 x 1.8 cm (shown in white arrow); 2- Fistula measuring about 1.2 x 0.6 cm (shown in black arrow)

On the right side, a smaller defect measuring about 1.2 × 0.6 cm was seen, extending anteroposteriorly from the distal aspect of tooth 11 to the distal surface of the canine, and mediolaterally from the midpalatal region to about 1 cm short of the free gingival margin (Figure 2).

A decision of closing the anterior palatal fistula using an anterior-based pedicled tongue flap was made. Informed written consent was obtained from the patient.

Treatment procedure

**Figure 3:**
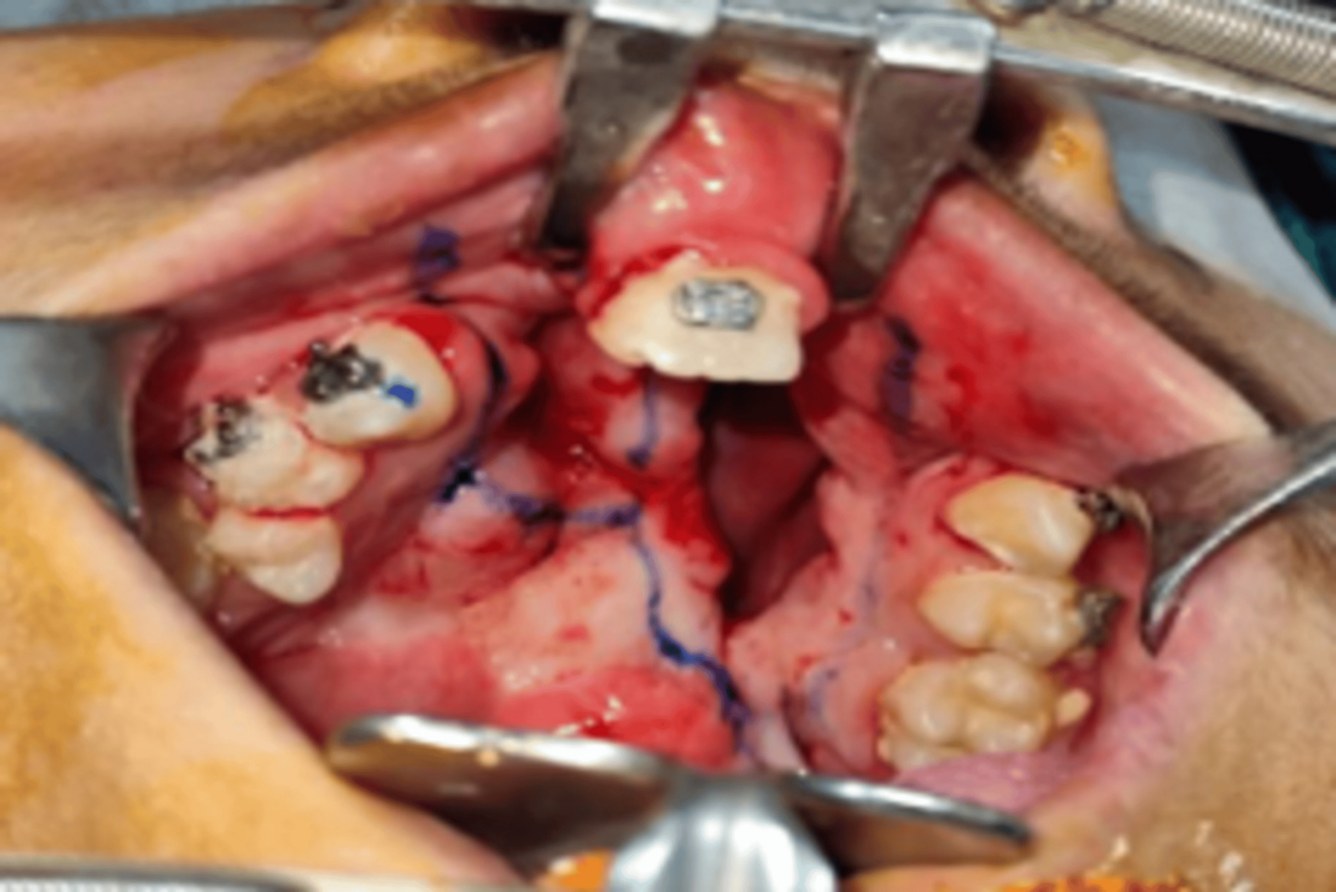
Intraoral marking around the fistula for incision

Standard blood tests, including complete blood count, bleeding time, and clotting time, were performed, and chest X-rays and 2D-ECHO were advised. All values were under normal limits, and orotracheal intubation was done under sterile aseptic conditions. Betadine painting and draping were done, and local infiltration was given with 2% lignocaine HCL with 1:80,000 adrenaline units. Intraoral markings were done for incision (Figure 3).

**Figure 4:**
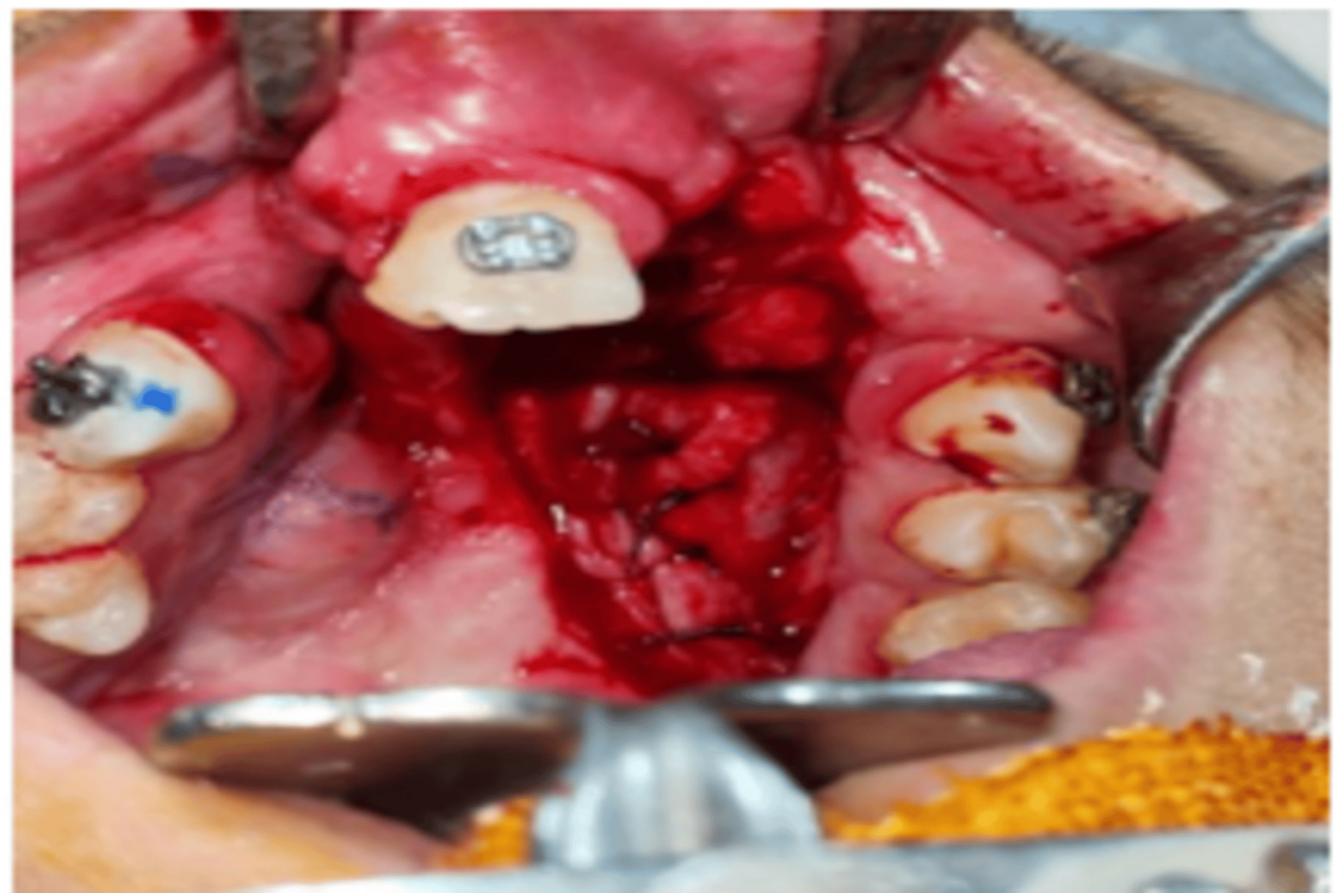
Picture showing closure of nasal mucosa by using vicryl sutures

An incision was made along the fistula margins, followed by careful dissection of the palatal and nasal mucosa. The nasal mucosa was approximated and sutured by using 3-0 Vicryl sutures (Figure 4).

**Figure 5:**
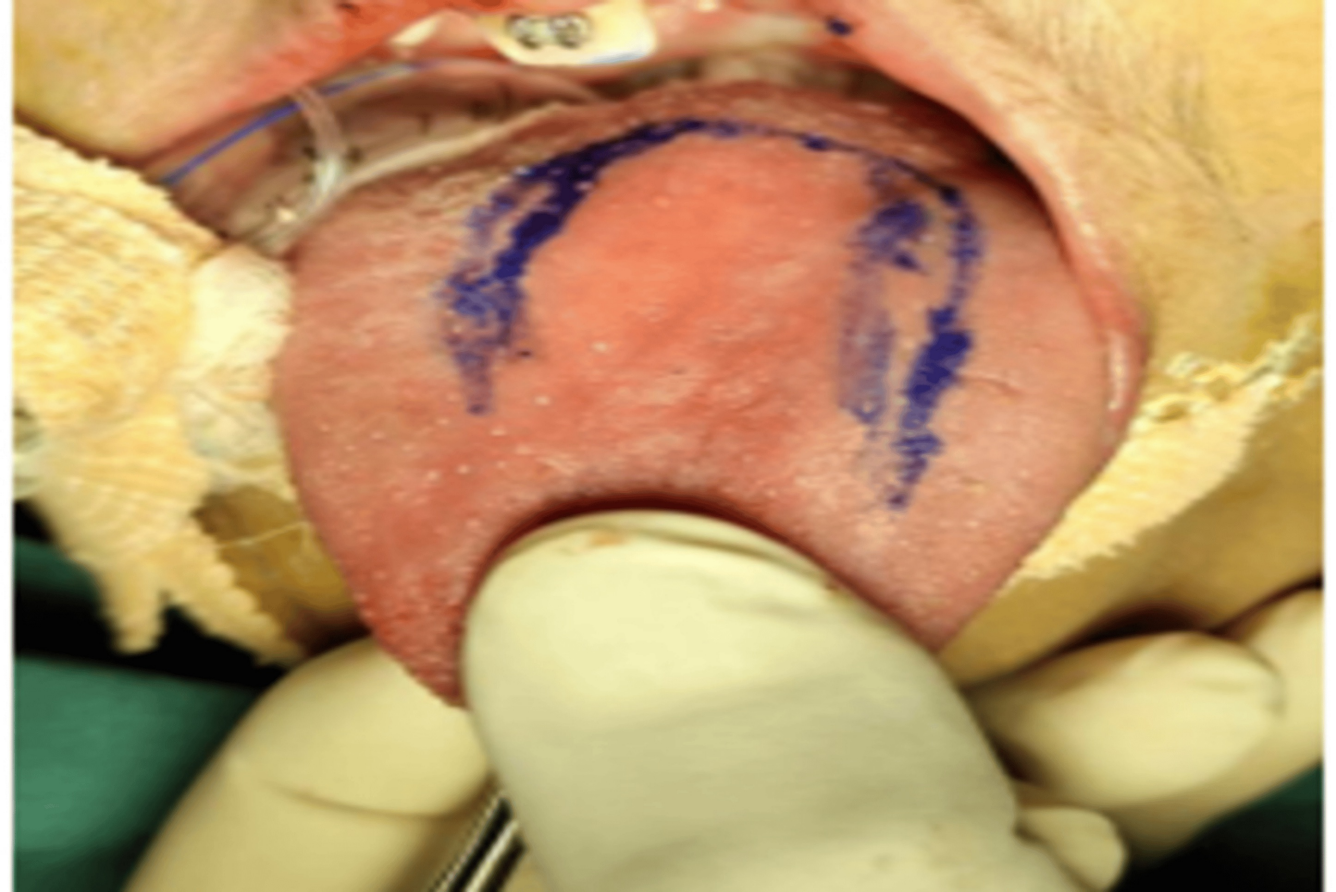
Picture showing marking on the anterior portion of tongue for incision

The anterior portion of the tongue was grasped with a Babcock forceps to facilitate proper retraction. Following the administration of local anesthesia, surgical markings were made on the tongue for incision (Figure 5).

**Figure 6:**
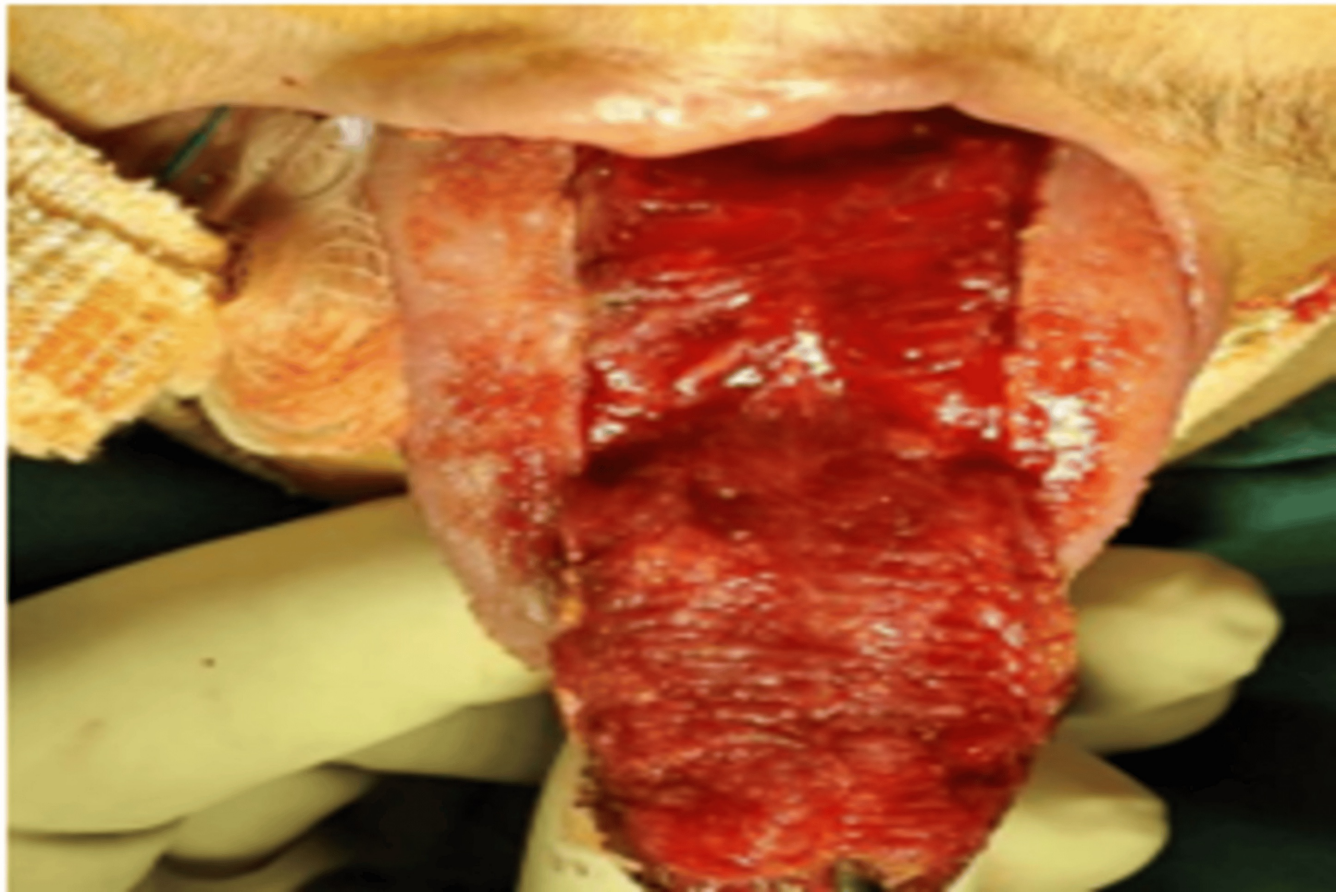
A tongue flap comprising mucosa along with longitudinal and transverse muscle layers was raised from the dorsal surface of the tongue

A surgical flap, slightly larger than the dimensions of the fistula, was carefully elevated. This flap included mucosa along with underlying longitudinal and transverse muscle components. It was designed with an anteriorly based pedicle to maintain adequate blood supply. The flap was prepared to ensure tension-free closure of the defect (Figure 6).

**Figure 7:**
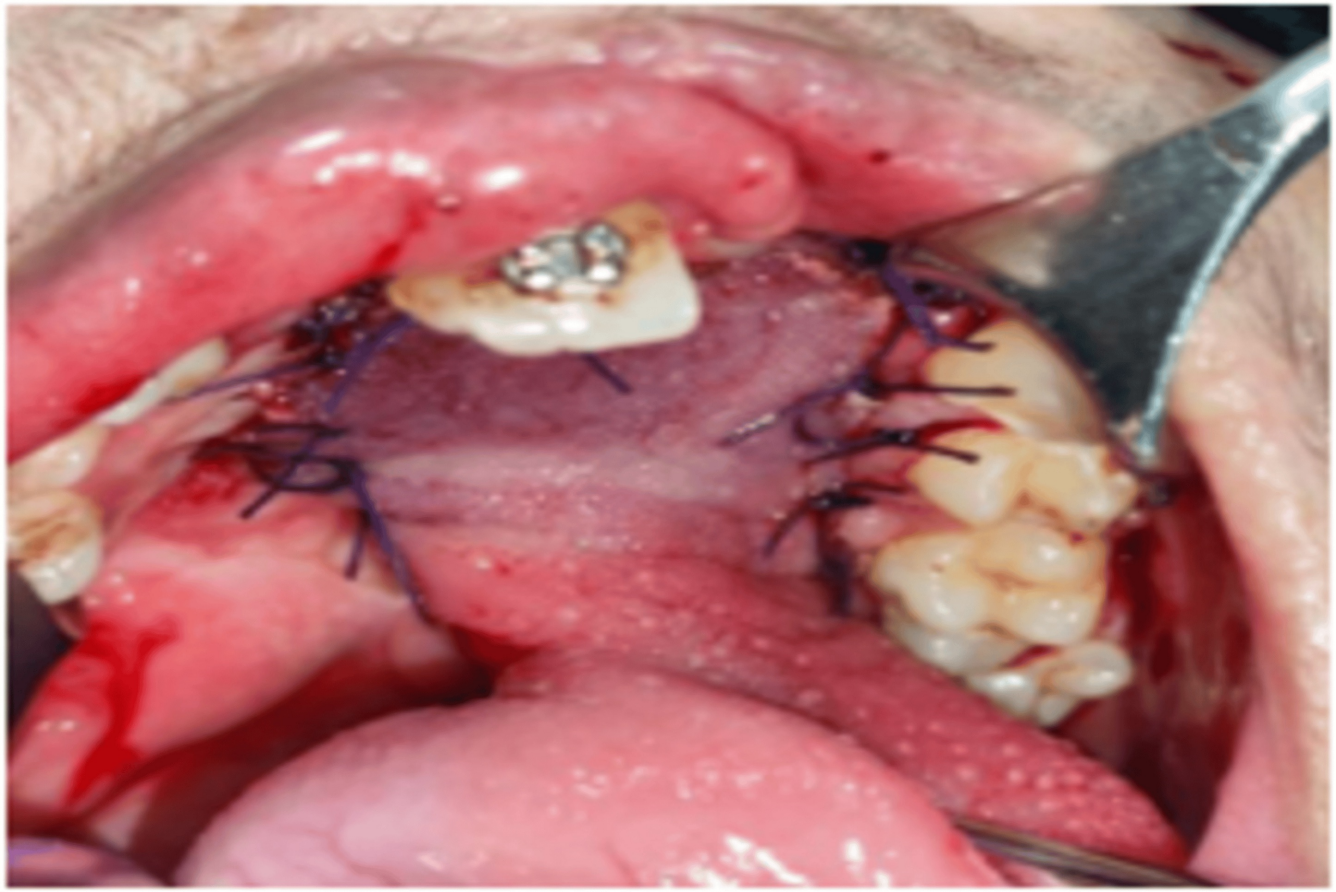
Picture showing tongue flap secured to the palate by using simple interrupted Vicryl sutures

The elevated tongue flap was carefully positioned to correspond with the palatal mucosal edges and was securely anchored to the adjacent mucosa using interrupted sutures, and one suture was placed in such a way that the palatal and nasal mucosa was included to prevent dead space, thus ensuring a stable and tension-free closure of the oronasal defect (Figure 7).

The patient was maintained on a liquid diet for the first week. Intermaxillary fixation was applied for three weeks, during which nutritional support was provided via a Ryle’s tube. The fixation was intermittently released for irrigation of the surgical site to promote optimal healing.

Post-operative care

During the postoperative phase, the patient was placed on a liquid diet, which was to be maintained until flap detachment. Both the patient and parents were clearly instructed to avoid excessive mouth opening, and a follow-up was scheduled in two to three weeks for flap division.

Flap separation

**Figure 8:**
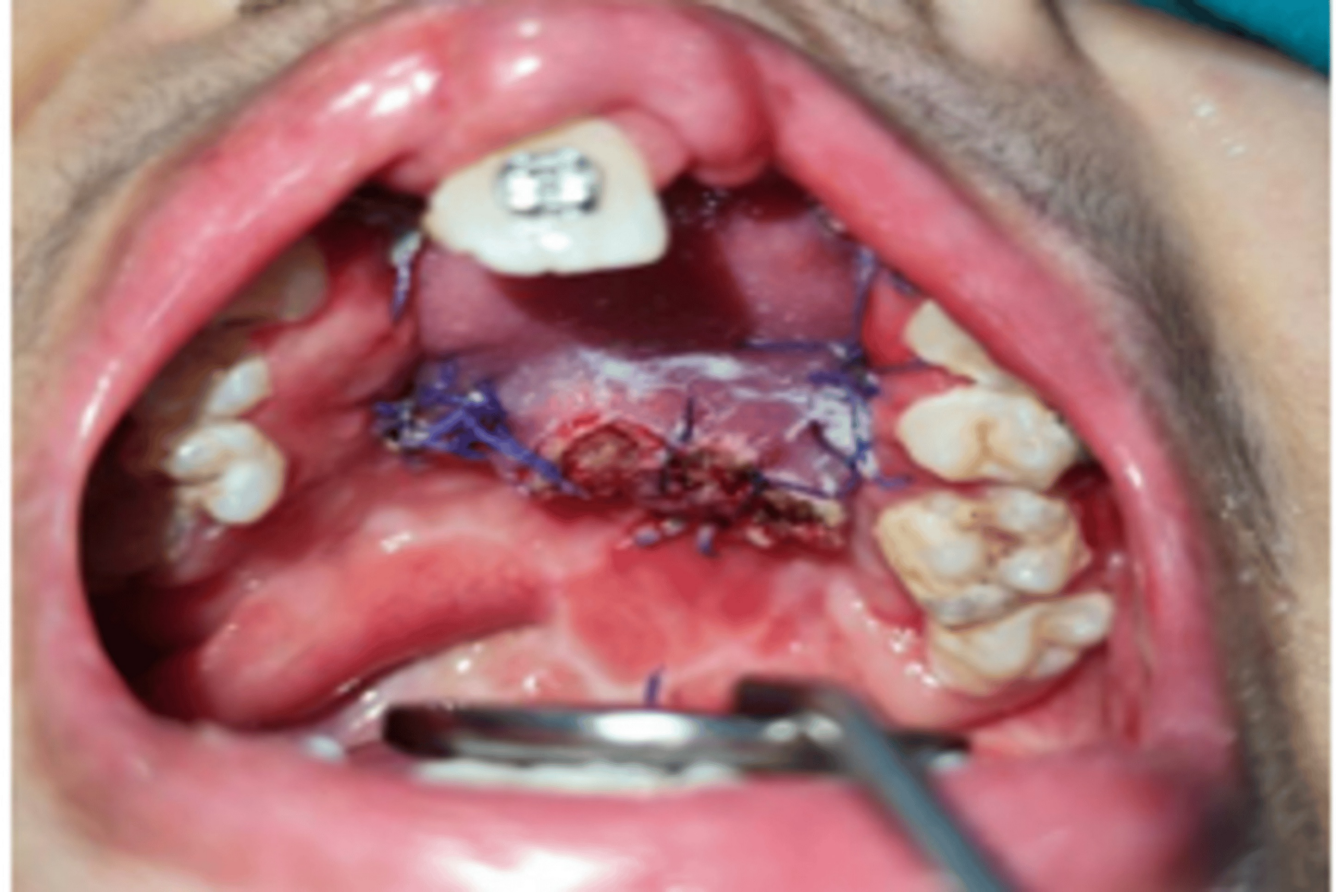
Pedicle division of tongue flap was done by using cautery

The second stage surgery was done after three weeks. After ensuring flap viability by giving 2% lignocaine HCL with 1:80,000 adrenaline units as infiltrations, the flap pedicle division was done using diathermy (Figure 8).

**Figure 9:**
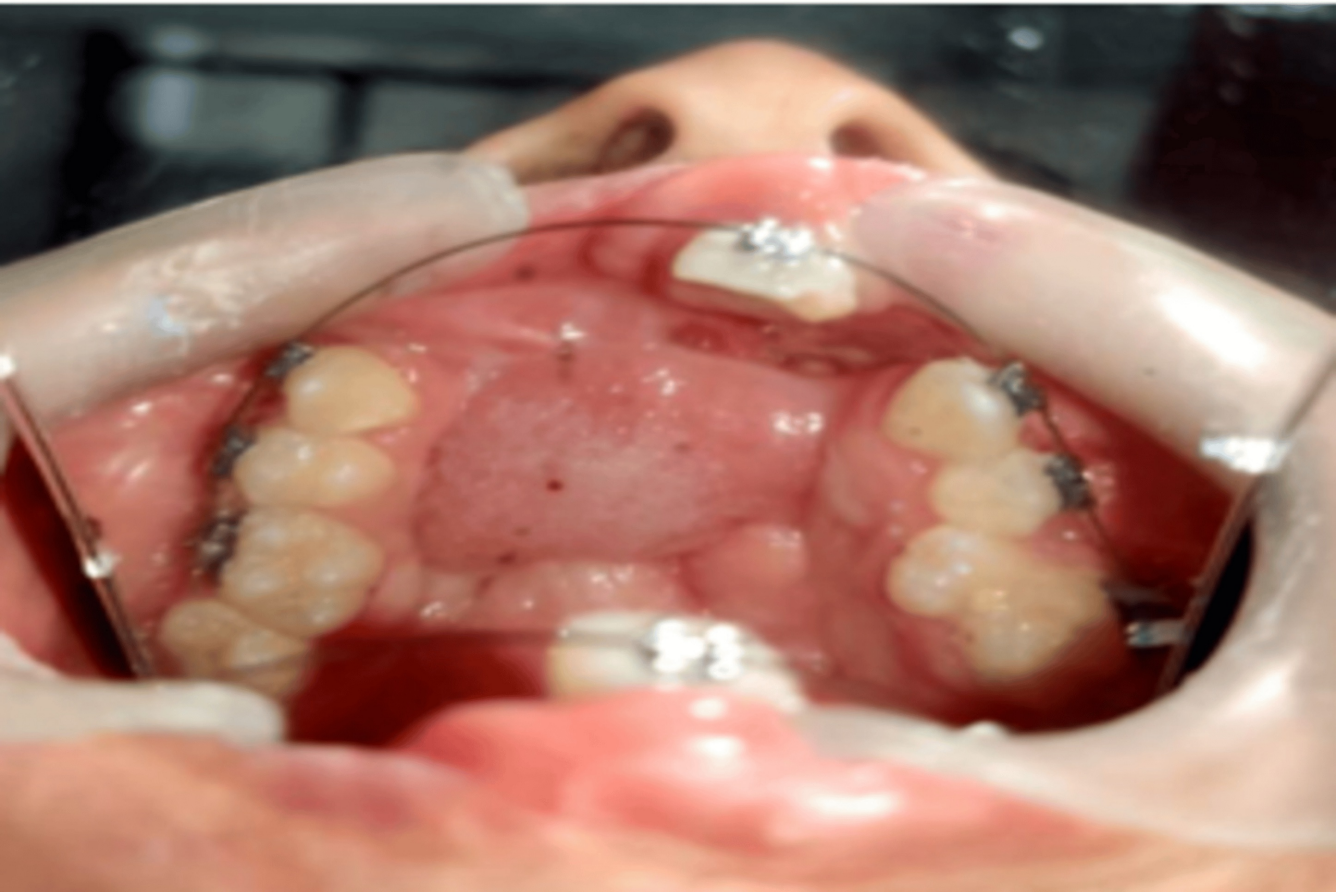
At 2 months recall, uneventful healing and closure of anterior palatal fistula

Oral fluid is allowed from the night of operation; soft diet from 4th to 5th day, then regular diet is started after one week. At the three-week follow-up, the donor site had healed completely, showing mild narrowing of the tongue but with preserved mobility and normal speech. At the two-month recall, the anterior palatal fistula exhibited successful closure with no postoperative complications (Figure 9).

## Discussion

The use of the tongue as a flap for oral cavity reconstruction was first introduced by Eiselsberg [4]. The anteriorly based dorsal tongue flap was introduced by Guerrero-Santos and Altamirano in 1966 for the surgical closure of a large palatal fistula [5]. The excellent versatility of the tongue flap can be attributed to its robust vascularization, primarily supplied by the lingual artery and its branches. Additionally, the ease of accessing tissue from the tongue allows for efficient closure of large palatal defects [6]. The tongue flap procedure is not associated with significant donor morbidity, with no impact on speech or movement; however, a temporary loss of taste sensation was observed [7].

Gupta et al. [8] considered it a successful procedure due to its ability to provide substantial amounts of well-vascularized tissue for fistula closure, while minimizing functional and aesthetic complications. Additionally, several other techniques mentioned in the literature are reviewed. Nakakita et al. [9] closed the palatal fistula by the use of a buccal musculomucosal flap.

Kuran et al. [10] utilized a three-layer flap consisting of a buccinator musculomucosal transposition flap for the nasal lining, a cranial bone graft for the palatal bone, and a local mucoperiosteal transposition flap for oral closure. The flaps and bone integrated effectively with the fistula.

Many authors have reported successful outcomes using free flap closure for palatal fistulas. Ninkovic et al. [11] utilized a free flap based on the dorsalis pedis and first dorsal metatarsal artery.

Schwabegger et al. [12] utilized osseous angular scapular flaps, while Krimmel et al. [13] used a laterally based upper arm flap with prelaminated mucosal lining. However, these techniques result in additional wounds in other areas of the body, increasing the postoperative discomfort and burden for the child.

Among the various reconstructive options available for palatal fistula closure, the anteriorly based pedicled tongue flap stands out due to its reliable vascularity, ease of harvesting, and excellent tissue compatibility with the palatal mucosa. Unlike free flaps such as the dorsalis pedis-first dorsal metatarsal artery flap and the osseous angular scapular flap, which require microvascular expertise and are more invasive, the tongue flap is a technically simpler, two-stage procedure with minimal donor site morbidity.

Compared to the laterally based upper arm flap with prelaminated mucosal lining, which may offer mucosal-like surface but involves complex planning and a distant donor site, the tongue flap provides similar mucosal texture, better proximity, and rapid integration. While musculomucosal flaps may be useful in small fistulae, they often lack the bulk and reach required for larger anterior palatal defects.

The anteriorly based tongue flap has proven to be a reliable and effective technique for the closure of large or recurrent anterior palatal fistulae, particularly when local tissues are scarred or insufficient for repair. This flap provides well-vascularized tissue with adequate bulk and flexibility, making it suitable for covering large defects and promoting mucosal integration. Elevation of the flap from the dorsal surface of the tongue ensures the inclusion of mucosa and underlying muscle layers, offering both structural support and durability. The anterior pedicle allows for easy rotation and alignment with anterior palatal defects. Overall, the pedicled tongue flap remains a preferred and time-tested technique, particularly for anterior fistulae, due to its adaptability, rich blood supply from the lingual artery, and predictable healing outcomes.

## Conclusions

Although it involves a two-stage surgical approach and may lead to temporary discomfort, the tongue flap continues to be the preferred technique for managing anterior palatal fistulas due to its reliability and effectiveness. The anteriorly based tongue flap is a reliable and effective technique, consistently yielding positive outcomes in the closure of anterior palatal fistulae. Despite its effectiveness, this approach requires a two-stage procedure - initial flap adaptation and later pedicle division - which demands patient compliance, especially in younger individuals. Postoperative care is crucial to prevent dehiscence, with emphasis on maintaining oral hygiene, limiting mouth opening, and adhering to dietary restrictions. Overall, the anteriorly based tongue flap remains a valuable option in challenging fistula closures where conventional methods have failed or are not feasible.
